# Assessing Cardiovascular Risk Among Polish Soldiers: Insights Using the POL SCORE Tool

**DOI:** 10.3390/jcm14062130

**Published:** 2025-03-20

**Authors:** Magdalena Zawadzka, Justyna Marszałkowska-Jakubik, Ewelina Ejchman-Pac, Beata Pająk-Tarnacka, Paweł Szymański

**Affiliations:** 1 Department of Epidemiology and Public Health, Medical University of Lodz, Żeligowskiego 7/9, 90-752 Lodz, Poland; 2 Department of Hygiene and Epidemiology, Military Institute of Hygiene and Epidemiology, Kozielska 4, 01-163 Warsaw, Poland; justyna.jakubik@wihe.pl (J.M.-J.); ewelina.e-pac@wihe.pl (E.E.-P.); 3 Department of Medical Biology, Military Institute of Hygiene and Epidemiology, Kozielska 4, 01-163 Warsaw, Poland; bepaj@wp.pl; 4 Department of Pharmaceutical Chemistry, Drug Analyses and Radiopharmacy, Medical University of Lodz, Muszyńskiego 1, 90-151 Lodz, Poland; pawel.szymanski@umed.lodz.pl; 5 Department of Radiobiology and Radiation Protection, Military Institute of Hygiene and Epidemiology, Kozielska 4, 01-163 Warsaw, Poland

**Keywords:** cardiovascular diseases, risk assessment, SCORE program, risk factors, public health, polish military

## Abstract

**Background/Objectives**: Maintaining the health and operational readiness of military personnel is a strategic priority, particularly in the context of cardiovascular diseases (CVDs), which remain a significant public health challenge in Poland. Despite a decline in mortality rates between 2006 and 2012, Poland continues to report higher premature mortality rates compared to the OECD average. This study highlights the importance of effective risk assessment and management strategies, employing the POL SCORE scale, an adaptation of the European Society of Cardiology’s Systematic Coronary Risk Evaluation (SCORE) project. **Methods**: This study included 196 participants, comprising soldiers and civilian employees of the Ministry of National Defense, to assess their 10-year cardiovascular mortality risk. Data were collected using clinical evaluations and self-reported questionnaires. **Results**: Findings revealed that 66.3% of participants were at moderate risk, with significant differences observed based on gender and education level. Notably, the average triglyceride level was 219.3 ± 114.31 mg/dL in the very high-risk group, compared to 97.4 ± 41.31 mg/dL in the low-risk group. Stress, reported by 88.2% of participants, emerged as the most prevalent work-related risk factor. Alarmingly, a lack of awareness regarding cardiovascular risk factors was observed, particularly among high-risk individuals. **Conclusions**: This study underscores the need for targeted health education, regular preventive screenings, and psychological support, particularly among military personnel. These interventions are crucial to mitigating the burden of CVDs and ensuring the operational readiness of armed forces.

## 1. Introduction

Myocardial infarction and stroke are two of the leading causes of death in Poland, making them a major societal concern. Myocardial infarction is the most severe consequence of coronary heart disease (CHD), while stroke is the most common condition among cerebrovascular diseases. In 2016, CHD and stroke were, respectively, the biggest and second-biggest causes of death in the country. Poland has a high-risk profile for cardiovascular disease (CVD), and in recent years, policy makers have recognized the need for action on CVDs and stroke as policy priorities. Over the past two decades, efforts to improve prevention and treatment have resulted in significant successes. Between 2006 and 2012, the standardized death rate from heart attacks dropped by 30%, and the standardized death rate for stroke decreased by 18% [1]. However, despite these advancements, many people still die prematurely due to heart attacks or strokes, highlighting the need for better long-term risk factor management to improve outcomes. Rates of premature mortality for heart attack and stroke are still significantly higher in Poland than the average for countries in the Organization for Economic Co-operation and Development (OECD). On average, certain risk factors are also more prevalent in Poland than in other OECD countries. Additionally, demographic and epidemiological changes are likely to increase the burden of these diseases in the future.

Assessing cardiovascular risk is crucial to preventing cardiovascular disease as it helps determine the potential benefits of using preventive interventions such as statins and aspirin for individual patients. The current guidelines suggest specific algorithms that use traditional risk factors such as blood pressure, lipids, and smoking, along with age, sex, and other factors to assess the risk [2]. In 2003, the European Society of Cardiology (ESC) initiated the Systemic Coronary Risk Estimation (SCORE) project to develop a risk scoring system for use in the clinical management of cardiovascular risk in European clinical practice [3]. The project brought together data from 12 European cohort studies, which were conducted mainly in general population settings. The studies included 2.7 million person-years of follow-up, involving 208,178 individuals (88,080 women and 117,098 men). The data showed that there were 7934 cardiovascular deaths, of which 5652 were due to coronary heart disease. The project used a Weibull model to calculate the 10-year risk of fatal cardiovascular disease, where age was considered a measure of exposure time to risk rather than a risk factor. The estimation equations were calculated separately for coronary heart disease and non-coronary cardiovascular disease and for high-risk and low-risk regions of Europe. Two parallel estimation models were developed based on total cholesterol and total cholesterol/HDL cholesterol ratio. The risk estimations were displayed graphically in simple risk charts. To examine the predictive value of the risk charts, they were applied to individuals aged 45–64, and the areas under ROC curves ranged from 0.71 to 0.84. As a result of the SCORE project, an algorithm estimating the risk of cardiovascular disease in people between 40 and 65 years has been created and widely distributed in clinical practice [3]. ESC experts have developed two versions of SCORE tables—one for low-risk and one for high-risk countries [4]. Countries such as Poland, the Czech Republic, Slovakia, and Hungary were included in the high-risk region [4].

Poland’s Cardiovascular Disease Prevention Program was designed and launched in 2002 in Łódź as a pilot program. In 2004, the program was implemented nationwide based on a decision by the President of the National Health Fund regarding the terms of concluding and implementing contracts for preventive health programs. The program aimed at early detection and modification of cardiovascular risk factors in the general population.

Since 2007, calibrated tables have been used in Poland to assess overall cardiovascular risk in the Polish population [5]. The risk was rated higher compared to the tables prepared by ESC experts for high-income countries. These SCORE tables were based on data from the NATPOL III study conducted in 2002. Since then, mortality rates due to cardiovascular diseases in Poland have decreased significantly. In 2002, the standardized mortality rate was 413.9/100,000, and by 2011 it had decreased to 317.8/100,000 (a decrease of 23.2%) [6]. This decline can be attributed to better control of risk factors and advances in diagnosing and treating cardiovascular diseases.

Based on current mortality rates due to cardiovascular diseases in Poland and the current prevalence of major risk factors in the Polish population, an update was made in 2015 [6]. The most common CV risk factors are excess waist circumference, dyslipidemia, smoking and hypertension [7]. Additionally, due to increased life expectancy in Poland and the need to assess cardiovascular risk in individuals over 65, the age range in the updated tables was extended for patients for whom risk can be calculated. The new POL SCORE tables allow cardiovascular risk calculation of in individuals aged 40 to 70 years [6]. This update reflects the evolution of preventive strategies, adjusting risk assessment tools to changing epidemiological and demographic trends.

It is important to note that ESC recommendations [4] suggest that risk analysis using algorithms and SCORE tables should be used for primary prevention in patients without a history of cardiovascular events or vascular diseases. Patients who have experienced cardiovascular events or have documented heart or vascular diseases are at very high risk and should be included in this group. The SCORE tables can also be used to assess cardiovascular risk in individuals with type 2 diabetes or chronic kidney disease (with GFR < 60 min/1.73 m^2^). These patients can be immediately assigned to the appropriate risk groups [6].

The POL SCORE algorithm is a widely used in clinical practice and epidemiological studies. It stratifies of patients based on their 10-year risk of fatal cardiovascular events, enabling healthcare professionals to tailor preventive strategies more effectively. By incorporating population-specific data, the POL SCORE provides a more accurate risk assessment for the Polish population than general European models. This specificity makes it an essential component in guiding clinical decision making and public health interventions to reduce cardiovascular disease burden [5].

Tools like the POL SCORE enable more precise identification of individuals at increased risk, allowing for the implementation of appropriate preventive interventions. The ultimate goal is to reduce the incidence of cardiovascular diseases and improve the quality of life for the population.

## 2. Materials and Methods

This research was conducted in accordance with the Declaration of Helsinki of the World Medical Association and was approved by the Bioethics Committee of the Military Medical Commission in Warsaw (Resolution No. 11/23). Participants were informed about the details of this study, its purpose and benefits of participation. All volunteers gave written informed consent to participate in this study and completed an original questionnaire assessing the state of knowledge of the soldier/military employee about cardiovascular diseases and risk factors for their occurrence.

The POL SCORE (Systematic Coronary Risk Evaluation) scale, calibrated for the Polish population, was used to assess the overall 10-year risk of cardiovascular death. For this purpose, each examined person had their blood pressure measured and lipid profile determined. Risk was defined based on age, gender, smoking, systolic blood pressure, and total cholesterol levels. The following risk classes were distinguished: very high: ≥10%; high: ≥5% and <10%; moderate: ≥1% and <5%; and low: <1%.

Blood pressure was measured by qualified medical personnel using an OMRON M7 Intelli IT device. Between October 2023 and February 2024, blood samples were collected from 196 soldiers and civilian military personnel in Polish military units. The collected blood samples were stored at 2–8 °C and sent to the laboratory for lipid profile testing.

This study aimed to assess the overall 10-year risk of cardiovascular death and to assess the self-awareness of the subjects regarding the risk factors for cardiovascular disease.

### Statistical Analysis

The collected material was analyzed statistically using Excel tools and Statistica 13.3. Descriptive statistics and parametric and non-parametric data analysis methods were used. The assessment of normality distribution was performed using the Shapiro–Wilk test. The Mann–Whitney U test was used to assess the relationship between quantitative variables, in the absence of normal distribution. The Kruskal–Wallis H test and post hoc analysis: multiple comparisons of mean ranks for all samples were used to analyze more than two groups of an ordinal variable. The relationship between two qualitative variables was assessed using the Pearson Chi^2^ test of independence. The correlation coefficient was calculated to express the interdependence between two variables. The r-Pearson correlation coefficient was used to describe the correlation between quantitative variables, and the Spearman rank correlation coefficient was used in the absence of normality distribution. A cluster analysis was performed using the k-means method and divided into POL SCORE risk categories. For all analyses, the significance level was assumed to be *p* ≤ 0.05.

## 3. Results

The study group consisted of 196 people, including 153 soldiers (78.1%) and 43 civilian employees of the Ministry of National Defense (21.9%). Most participants, i.e., 71.4% (*n* = 140), were men. The study group was aged 40–67 years ($\bar{x}$ = 7.83 ± 5.32; Q1 = 44.00 and Q3 = 51.00). More than half of the participants, i.e., 58.7% (*n* = 115), had higher education, 38.3% (*n* = 75)—secondary education, the rest—vocational education. A total of 84.5% (*n* = 164) of participants were married.

According to the POL SCORE, 2/3 of participants (66.3%; *n* = 131) calculated the 10-year risk of death from cardiovascular causes as moderate (≥1% and <5%) more than every fifth participants (21.4%; *n* = 42) as high (≥5% and <10%), in 3.6% (*n* = 7) as very high (≥10%), in the remaining low (<1%). A detailed analysis of the participants taking into account the POL SCORE risk classes is presented in Table 1.

A statistically significant relationship was obtained in the group of subjects divided into POL SCORE risk groups and gender (Z = 6.459; *p* = 0.0000) and a moderate correlation between the above variables (rho = 0.463; *p* < 0.0500). Also, the result was statistically significant among soldiers and civilian employees of the Ministry of National Defense (Z = 2.819; *p* = 0.0048), but the correlation between variables was weak (rho = 0.242; *p* = 0.0006). The analysis indicated a statistically significant relationship between the military service corps and individual POL SCORE risk groups (H = 12.170; *p* = 0.0325); however, post hoc analysis of multiple comparison of mean ranks for all samples showed only slight differences between the groups: non-commissioned officer, senior officer, and general, admiral. In addition, the average blood concentration of total cholesterol, LDL and triglycerides (TG) increases with the POL SCORE risk category, 212.6–258.6 mg/dL, 127.8–158.3 mg/dL and 97.4–219.3 mg/dL, respectively. A similar relationship was noted in the systolic blood pressure value, i.e., 131.7–157.0 mmHg. In turn, with the increase in the POL SCORE risk category, the average blood HDL concentration decreases—67.1–57.6 mg/dL.

Among people whose family members suffer from cardiovascular diseases, 68.9% are participants with moderate risk, according to the POL SCORE. Almost every fifth participants (17.9%) with high or very high risk, according to the POL SCORE, has no knowledge on this subject (Table 2). A statistically significant difference was found between the study groups (H = 6.972; *p* = 0.0306); however, post hoc analysis, multiple comparison of mean ranks for all samples, showed only slight differences between the study groups. Moreover, no relationship was observed between the occurrence of cardiovascular diseases in the family and the concentration of HDL in the blood of the subjects (H = 0.595; *p* = 0.7425), as well as the level of HDL in the blood and the POL SCORE risk category (H = 5.609; *p* = 0.1322).

A total of 50% of participants diagnosed with cardiovascular disease have a moderate risk, according to the POL SCORE. A total of 2/3 of participants who have not been diagnosed with cardiovascular disease have a moderate risk of death according to the POL SCORE, and ¾ who have no knowledge on this subject (Table 3). The analysis showed a statistically significant difference between the study groups (H = 8.080; *p* = 0.0176); however, post hoc analysis, multiple comparison of mean ranks for all samples showed only slight differences between the study groups. Moreover, cluster analysis by POL SCORE risk categories and the presence of selected comorbidities showed only 15.7% of the examined (*n* = 30) to have hypertension, including 9.9% (*n* = 19) with moderate risk of cardiovascular death, 5.23% (*n* = 10) with high risk and 0.52% (*n* = 1) with very high risk. Atherosclerosis and ischemic heart disease occurred in single persons with a moderate POL SCORE risk, i.e., 1.57% (*n* = 3) and 0.53% (*n* = 1).

**Figure 1:**
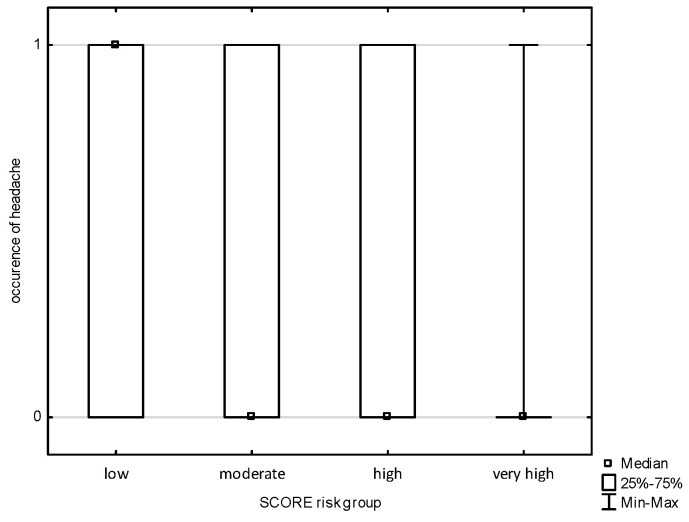
The occurrence of headaches depending on the POLSCORE risk group.

**Figure 2:**
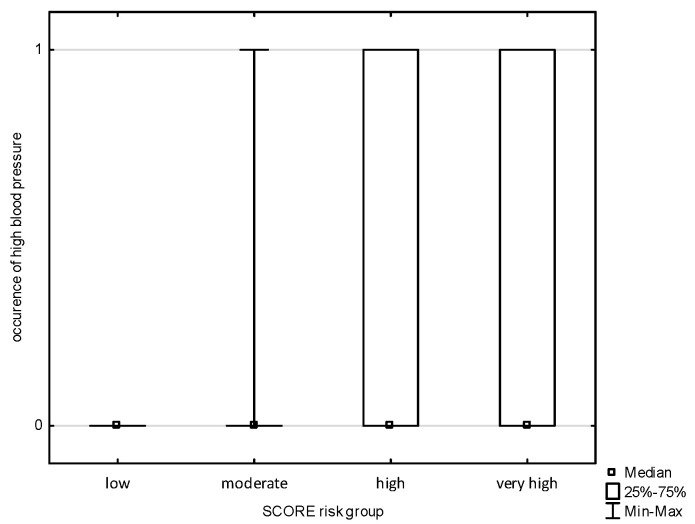
The occurrence of increased blood pressure depending on the POLSCORE risk group.

An analysis of the occurrence of symptoms of cardiovascular diseases in the subjects was carried out and divided into POL SCORE risk categories. There was no correlation in the study groups with chest pain, shortness of breath, palpitations, or pain in the lower limbs. The analysis allowed us to observe a statistically significant relationship (Figure 1 and Figure 2) between the POL SCORE risk groups and the occurrence of headache in the examined persons (H = 5.691; *p* = 0.0171) and increased blood pressure (H = 3.852; *p* = 0.0497). Post hoc multiple-comparison analysis of mean ranks for all samples showed only slight differences between the study groups.

According to the POL SCORE, most people (62.9%) who assessed their health as very good have moderate risk. According to the POL SCORE, every third participants who assessed their health as satisfactory has high or very high risk (Table 4). The analysis did not show a statistically significant relationship in the assessment of health status in individual POL SCORE risk groups (H = 0.577; *p* = 0.9655) and in the group of soldiers and civilian employees of the Ministry of National Defense (Chi^2^ = 7.573; df = 4; *p* = 0.10851). A statistically significant relationship was observed between the participants’ health assessment and the military service corps (Chi^2^ = 44.350; df = 20; *p* = 0.00135).

Every third participants with a high risk of death, according to the POL SCORE, undergoes preventive examinations once a year. In turn, 26.1% of participants whose 10-year risk of cardiovascular death is at least 5% are tested once every five years (Table 5). The analysis showed a statistically significant relationship in the frequency of preventive tests performed by participants in particular POL SCORE risk groups (H = 17.005; *p* = 0.0045). In turn, no significant differences were observed in the group of soldiers and civilian employees of the Ministry of National Defense (Chi^2^ = 3.824; df = 5; *p* = 0.57495) and in the division into the military service corps (Chi^2^ = 16.097, df = 25; *p* = 0.91190).

The question included in the survey checked the participants’ self-awareness regarding the occurrence of risk factors for cardiovascular diseases (Table 6). According to the majority of the participants, they do not have any risk factors for cardiovascular diseases. Stress was the most frequently indicated predictor, followed by a poor diet, a positive family history, and being overweight. Analysis of all the factors mentioned indicated a statistical relationship only in overweight, low physical activity, smoking, and drinking alcohol in the group of soldiers and civilian workers. An analysis of the occurrence of risk factors for cardiovascular diseases in the participants was also conducted and divided into POL SCORE risk categories. The analysis allowed us to observe a statistically significant relationship between the POLS SCORE risk groups and the occurrence of elevated blood cholesterol in the participants (H = 11.760; *p* = 0.0082) and smoking (H = 16.131; *p* = 0.0011). Post hoc analysis of multiple comparisons of mean ranks for all samples showed only slight differences between the groups studied.

Most participants indicated stress among the listed risk factors in the work environment (88.2%). A total of 89.1% of those exposed to stress were soldiers. A total of 44.6% were non-commissioned officers and 19.3 were senior officers. Every third participants (65.7%) exposed to stress had a moderate risk of death, according to the POL SCORE, and every fourth (22.3%) had a high risk. Approximately ¾ of participants stated that they were not exposed to low physical activity and improper nutrition in their work environment. Every fifth soldier (20.4%) declared low activity in the work environment, and every third (29.6%) had improper nutrition (Table 7). However, a statistically significant result was obtained only in the case of low physical activity (Chi^2^ = 14.056; df = 1; *p* = 0.0018). Analysis by military service corps also indicated statistically significant results only for low physical activity (Chi^2^ = 15.462; df = 5; *p* = 0.0856). No significant relationship was noted between any of the risk factors in the work environment and the POL SCORE risk groups.

In the group of participants with a high and very high risk of death according to the POL SCORE, the average level of exposure to stress in the work environment was most often indicated, i.e., 52.5% and 57.1%, respectively (Table 8). Every third participants with a significant level of stress was in the group with a high or very high 10-year risk of death from cardiovascular causes. The analysis did not show a statistically significant relationship in the degree of exposure to stress in individual POL SCORE risk groups (H = 7.767; *p* = 0.1005), in the group of soldiers and civilian employees of the Ministry of National Defense (Chi^2^ = 2.371; df = 4; *p* = 0.66774) and by military service corps (Chi^2^ = 27.116; df = 20; *p* = 0.13203).

## 4. Discussion

Using the POL SCORE scale, this study analyzed the 10-year risk of cardiovascular disease-related mortality among 196 soldiers and civilian employees of the Ministry of National Defense. The results indicate significant differences in risk levels depending on gender, education, and health status, emphasizing the importance of understanding risk factors in the context of public health.

The analysis reveals that the majority of participants (66.3%) have a moderate risk of cardiovascular disease-related mortality. It is noteworthy that a similar group was classified as having high risk (21.4%) and as being unaware of a family history of cardiovascular diseases (17.9%), a known risk factor. This underscores the need for intensified educational efforts regarding cardiovascular risk factors. Our findings on family history, indicating that 68.9% of participants with moderate risk have close relatives diagnosed with cardiovascular diseases, have profound implications for our understanding of risk factors. The lack of correlation between family history and serum HDL levels (H = 0.595; *p* = 0.7425) suggests that other factors may have a greater influence on participants’ lipid profiles.

Similar alarming findings were observed in the MIL-SCORE cross-sectional study by Gielerak et al., conducted between 2012 and 2016 on 6440 soldiers (97% male). The MIL-SCORE results suggest that Polish soldiers have multiple cardiovascular risk factors similar to those observed in the general population. Nearly half of the participants were current or former smokers (46%). A sedentary lifestyle was reported by almost one-third of individuals over the age of 40. The prevalence of hypertension in the subgroup over 50 years old was nearly 45%, and the prevalence of overweight and obesity reached 58% and 27%, respectively, in this group. Over half of the participants were found to have elevated total cholesterol levels, while the prevalence of abnormal low-density lipoprotein (LDL) cholesterol was even higher, reaching 60%. Elevated triglyceride levels were observed in 36% of soldiers, while low high-density lipoprotein (HDL) cholesterol and hyperglycemia were identified in 13% and 16% of participants, respectively [8]. Our study demonstrates that with increasing POL SCORE risk categories, the average blood concentrations of total cholesterol, LDL and triglycerides rise. A similar trend was observed for systolic blood pressure values. Conversely, HDL cholesterol levels decrease as the POL SCORE risk category increases.

The findings of the MIL-SCORE study revealed that the cardiovascular health of professional Polish soldiers mirrors the unfavorable trends observed in the general population. A high prevalence of obesity, metabolic disorders, and abnormal blood pressure was noted, highlighting the need for targeted interventions. The incidence of cardiovascular risk factors increases with age, and their cumulative effect places a significant number of individuals at risk of atherosclerotic events. Furthermore, the study showed that young adults who are physically active exhibit fewer cardiovascular risk factors. Healthy lifestyle habits, such as not smoking, maintaining a BMI below 25, and adhering to a balanced diet, were inversely associated with total cholesterol, blood pressure, BMI, triglycerides, lipids, and fasting glucose levels [8].

Incorporating data from the POL SCORE scale provides a deeper understanding of the mechanisms underlying these trends, emphasizing the importance of individual risk factors in the broader context of public health.

As early as 2008, Leigh K. McGraw observed an alarming increase in cardiovascular risk factors within the military population, particularly in the context of operations conducted in high-stress environments. This phenomenon affects even young adults, which is especially concerning given the need to maintain a fit and operationally ready military force. He identified hypertension in 29.5% and hypertriglyceridemia in 24.4% of the examined military personnel (*n* = 999) [9]. In our study, only 15.7% of the examined (*n* = 30) had hypertension, including 9.9% (*n* = 19) with moderate risk of cardiovascular death, 5.23% (*n* = 10) with high risk and 0.52% (*n* = 1) with very high risk. Additionally, it should be emphasized that 88.2% of participants indicated exposure to stress in the work environment. However, only 58.1% of participants consider stress as a risk factor for cardiovascular diseases.

Obesity is also a key risk factor for cardiovascular disease, with 14% of soldiers in our study classified as overweight. In the United States, the proportion of obese professional soldiers increased from 10.4% in 2012 to 21.6% in 2022 [10]. The highest obesity rates are reported in the US Navy and Marine Corps [11].

The statistical relationship between gender and POL SCORE risk classes (Z = 6.459; *p* = 0.000) indicates differences in health risk perception between men and women, highlighting the need to tailor preventive strategies to the specific needs of both genders.

In Gielerak’s study, 189 soldiers (3% of the group) were classified as being at high or very high cardiovascular risk. In the subgroup over 50 years old, high and very high cardiovascular risk levels were observed in almost one-third of soldiers. The relative risk assessed in younger subgroups was moderate or high [8]. As indicated by Huiban et al., the overall risk level, ranging from “moderate” to “very high,” affected one in five people according to the SCORE model [20.1% (18.6–21.6)], one in six according to the Framingham model [16.3% (14.9–17.7)], and nearly one in three according to the summation model. A study involving 2792 professional aircrew members showed that over two-thirds of this predominantly male population (86.2%) had no more than one cardiovascular risk factor [69.9% (68.2–71.6)]. In 82.5% of cases, the risk factor was lipid metabolism disorders, as per current European criteria [55.8% (52.4–59.1)] or smoking [26.7% (23.8–29.8)] [12]. Also in our study, a statistically significant correlation was observed between the POLS SCORE risk groups and the occurrence of elevated blood cholesterol levels in participants (H = 11.760; *p* = 0.0082) and tobacco smoking (H = 16.131; *p* = 0.0011).

Surprisingly, 62.9% of participants rated their health as very good despite many being classified in moderate or high-risk groups. This discrepancy between subjective health assessment and objective risk may indicate a lack of awareness regarding health status, which is concerning in the context of cardiovascular disease prevention. A 2017 study analyzed the overall health status of military personnel, with a particular focus on the relationship between health behaviors and psychosocial variables. The findings were consistent with ours and showed that subjective health assessments often exceeded objective indicators. This suggests that soldiers may not be fully aware of their actual health status [13].

Findings on the frequency of preventive examinations reveal that only one-third of high-risk participants undergo screenings once a year. This highlights the need to raise awareness about the importance of regular check-ups in preventing cardiovascular diseases, particularly in high-risk groups. Additionally, 17.4% of high-risk participants undergo preventive examinations only every five years.

The analysis of workplace risk factors identified stress as the most frequently reported factor, which is particularly significant in the context of military service. High levels of stress in high-risk groups (32.5% in the high-risk group and 28.6% in the very high-risk group) highlight the necessity of implementing psychological support programs and stress management strategies in the workplace. Numerous studies confirm that post-traumatic stress disorder (PTSD) is a major contributing factor to the development of cardiovascular diseases among soldiers and veterans, increasing the risk of conditions such as hypertension. PTSD is also positively associated with other cardiovascular risk factors, including elevated heart rate, smoking, dyslipidemia, and obesity [14].

In our study, the majority of participants identified stress as one of the key workplace risk factors (88.2%), with 89.1% of those exposed to stress being soldiers. One-third of participants exposed to stress (65.7%) were in the moderate-risk group for mortality according to the POL SCORE, while one-fourth (22.3%) were in the high-risk group.

Polish preventive programs aimed at soldiers include initiatives focused on stress management and the prevention of post-traumatic stress disorder (PTSD). These programs are designed to support mental health and mitigate the long-term effects of service in high-risk conditions. The Department of Military Health Services ensures that every soldier, officer, or employee whose health condition requires it has access to a 14-day therapeutic and preventive stay, including “anti-stress training,” in military spa and rehabilitation hospitals after completing service abroad [15].

Jennifer Xu’s data suggest that the occupational duties of tactical athletes (including military, law enforcement, firefighters, and emergency response providers) increase their risk of cardiovascular disease, possibly triggered by intense surges of physical activity and periods of high emotional stress. As the tactical athlete ages and becomes more sedentary, their risk factor profile resembles that of the general population. Occupational exposure to excessive noise and smoke, as well as suboptimal working conditions such a poor diet and lack of sleep have also been cited as risk [16].

In our study, 23.1% of participants reported smoking, while among soldiers, 19.6% smoked. In comparison, according to data published by McGraw, the overall smoking rate among military personnel was 32.2%, with the highest rates observed in the Army and Marine Corps, at 38.2% and 36.3%, respectively. These findings are consistent with the results of a 2002 US Department of Defense study on health behaviors among military personnel, which reported that 33.8% of personnel had smoked cigarettes in the past 30 days, and 32.6% had smoked within the past 12 months [10].

In summary, the findings of this study highlight the significant role of health education and the need to promote regular preventive screenings and psychological support to reduce cardiovascular disease risk among soldiers and civilian employees. While soldiers are commonly perceived as models of health and physical fitness, the data suggest that this group exhibits cardiovascular risk factors similar to those of the general population. Cardiovascular risk factors, including obesity among soldiers, pose not only a health issue but also a question of operational readiness, necessitating urgent measures to improve the situation. Further research and the development of preventive programs focused on early risk assessment and modification, including smoking cessation, stress management, and promoting of healthy dietary habits and lifestyles, are strongly recommended.

## 5. Conclusions

Based on the conducted research, several critical conclusions can be drawn regarding cardiovascular disease-related mortality risk among soldiers and civilian employees of the Ministry of National Defense. A significant finding was the high prevalence of moderate risk, with 66.3% of participants falling into this category. This suggests that a substantial portion of the population may require future health interventions, even if they do not currently qualify as high-risk individuals. The analysis also revealed significant gender differences in risk levels (Z = 6.459; *p* = 0.000), emphasizing the need for tailored preventive strategies that address the specific needs of men and women in this occupational group.

Another key finding was the low awareness of cardiovascular disease risk factors among high-risk individuals. Nearly one-fifth of participants were unaware of these factors, highlighting the necessity for enhanced health education to improve understanding and awareness of risks. Family history also emerged as a notable factor, with 68.9% of participants in the moderate-risk group reporting close relatives with cardiovascular diseases. While no direct correlation was found with serum HDL levels, this suggests that family history may still play an important role in risk assessment.

Preventive screenings were found to be infrequent, as only 33% of high-risk individuals undergo screenings once a year. This underscores the importance of promoting regular check-ups for early detection and risk management. Additionally, stress was identified as the most common risk factor, with 88.2% of participants experiencing it in the workplace. High stress levels may contribute to an increased risk of cardiovascular diseases, underlining the need for the implementation of psychological support programs.

This study also identified a discrepancy between subjective health assessments and objective risk levels. While 62.9% of participants rated their health as very good, many were classified into moderate or high-risk groups. This points to a lack of awareness regarding actual health status and emphasizes the importance of improving risk perception among individuals.

Cardiovascular risk factors were found to pose a serious threat to the operational readiness of soldiers. Although the military is often perceived as a group of young and physically fit individuals, cardiovascular risk becomes increasingly apparent with age. To address this issue, regular assessments and efforts to monitor and reduce cardiovascular disease risk should begin at the recruitment stage and continue throughout military careers, encompassing both active-duty and reserve personnel.

To more effectively reduce the risk of cardiovascular diseases in this occupational group, it is recommended to conduct longitudinal analyses examining the impact of proposed interventions such as stress management, health education, and regular screenings. Consideration should also be given to the development and implementation of pilot prevention programs that span all stages of a military career, from recruitment to retirement. The introduction of such measures should be supported by modern technologies to facilitate health monitoring and reminders about preventive care.

Finally, inter-institutional collaboration should be emphasized. Close cooperation between scientific institutions and the Department of Military Health Services is essential for the development, implementation, and evaluation of effective preventive programs. Such collaboration will enable better alignment of strategies with the needs of soldiers and defense personnel, while also supporting the transfer of knowledge and experience from research into military practice.

## Figures and Tables

**Table 1:** Characteristics of the participants divided into POL SCORE risk classes (* for *p* < 0.0000, ** for *p* < 0.0500, *** *p* = 0.0048, **** *p* = 0.0006, and ***** *p* = 0.0325).

Variable		POL SCORE Risk Class			
		Low < 1%	Moderate ≥ 1% and <5%	High ≥ 5% and <10%	Very High ≥ 10%
Gender [%] *	male **	-	66.4	28.6	5.0
	female **	28.6	67.8	3.6	-
Study group [%] ***	civilian worker ****	18.6	72.1	7.0	2.3
	soldier ****	5.2	65.4	25.5	3.9
Military service corps [%] *****	private soldier	12.5	62.5	25.0	-
	non-commissioned oficer	5.7	63.6	28.4	2.3
	junior oficer	5.0	70.0	20.0	5.0
	senior oficer	2.9	67.7	20.6	8.8
	general, admiral	-	66.7	33.3	-
	lack	18.6	72.1	7.0	2.3
total cholesterol [mg/dL]	min	172.2	121.0	167.0	185.6
	max	304.0	459.6	321.1	323.6
	average	212.6 ± 31.76	228.7 ± 46.45	240.6 ± 40.78	258.6 ± 44.85
systolic blood pressure [mmHg]	min	115.0	104.0	114.0	140.0
	max	145.0	170.0	190.0	189.0
	average	131.7 ± 9.02	135.7 ± 13.99	149.0 ± 17.59	157.0 ± 15.69
HDL [mg/dL]	min	39.0	31.0	31.0	33.0
	max	85.0	199.0	88.0	101.1
	average	67.1 ± 11.29	62.7 ± 20.79	61.5 ± 19.41	57.6 ± 19.13
LDL [mg/dL]	min	84.3	35.0	91.0	92.7
	max	205.0	353.6	207.8	182.0
	average	127.8 ± 28.95	141.6 ± 40.64	149.1 ± 33.01	158.3 ± 31.73
TG [mg/dL]	min	49.0	13.0	56.0	80.0
	max	192.0	597.0	608.0	383.9
	average	97.4 ± 41.31	150.4 ± 99.16	175.5 ± 104.38	219.3 ± 114.31

**Table 2:** Characteristics of the participants divided into POL SCORE risk classes and cardiovascular diseases in the family (H = 6.972; * *p* = 0.0306).

POL SCORE Risk Class	Cardiovascular Diseases in the Family *		
	Yes [%]	No [%]	I Don’t Know [%]
low (<1%)	11.1	4.1	7.1
moderate (≥1% and <5%)	68.9	60.3	75.0
high (≥5% and <10%)	16.7	31.5	14.3
very high (≥10%)	3.3	4.1	3.6

**Table 3:** Characteristics of the participants divided into POL SCORE risk classes and diagnosed cardiovascular diseases in the participants (H = 8.080; * *p* = 0.0176).

POL SCORE Risk Class	Diagnosed Cardiovascular Diseases in the Participants *		
	Yes [%]	No [%]	No Opinion [%]
low (<1%)	0	8.9	8.3
moderate (≥1% and <5%)	50.0	66.7	75.0
high (≥5% and <10%)	45.0	20.0	16.7
very high (≥10%)	5.0	4.4	0.0

**Table 4:** Characteristics of the participants divided into POL SCORE risk classes and assessment of health status (H = 0.577; * *p* = 0.9655).

POL SCORE Risk Class	Assessment of Health Status by Participants *				
	Very Good [%]	Good [%]	Satisfactory [%]	Badly [%]	No Opinion [%]
low (<1%)	11.4	7.3	9.1	0	5.6
moderate (≥1% and <5%)	62.9	66.4	59.1	100.0	72.2
high (≥5% and <10%)	20.0	23.6	22.7	0	22.2
very high (≥10%)	5.7	2.7	9.1	0	0

**Table 5:** Characteristics of the participants divided into POL SCORE risk classes and frequency of preventive examinations performed (H = 17.005; * *p* = 0.0045).

POL SCORE Risk Class	Frequency of Preventive Examinations Performed by Participants *				
	More Than Once a Year [%]	Once a Year [%]	Once Every 5 Years [%]	When I Have Symptoms [%]	I Don’t Do It [%]
low (<1%)	-	1.9	8.7	20.0	15.7
moderate (≥1% and <5%)	90.9	55.8	65.2	66.7	72.7
high (≥5% and <10%)	9.1	34.6	17.4	13.3	11.0
very high (≥10%)	-	7.7	8.7	-	0.6

**Table 6:** Characteristics of participants divided into study groups and presence of risk factors for cardiovascular disease.

Risk Factor		Study Group		Total [%]
		Soldier [%]	Civilian Worker [%]	
overweight	Yes	30.1	3.8	33.9
	No	48.9	17.2	66.1
Statistics	Chi^2^ = 5.585; df = 1; *p* = 0.01811			
obesity	Yes	14.0	3.2	17.2
	No	65.1	17.7	82.8
Statistics	Chi^2^ = 0.114; df = 1; *p* = 0.73484			
poor diet	Yes	34.4	8.6	43.0
	No	44.6	12.4	57.0
Statistics	Chi^2^ = 0.079; df = 1; *p* = 0.77820			
high blood cholesterol	Yes	15.1	4.8	19.9
	No	64.0	16.1	80.1
Statistics	Chi^2^ = 0.314; df = 1; *p* = 0.57522			
type 2 diabetes	Yes	1.6	0.6	2.2
	No	77.4	20.4	97.8
Statistics	Chi^2^ = 0.040; df = 1; *p* = 0.84127			
low physical activity	Yes	14.5	8.6	23.1
	No	64.5	12.4	76.9
Statistics	Chi^2^ = 8.903; df = 1; *p* = 0.00285			
smoking	Yes	21.0	2.1	23.1
	No	58.1	18.8	76.9
Statistics	Chi^2^ = 4.592; df = 1; *p* = 0.03210			
alcohol drinking	Yes	18.3	1.1	19.4
	No	60.7	19.9	80.6
Statistics	Chi^2^ = 6.398; df = 1; *p* = 0.01142			
stress	Yes	45.2	12.9	58.1
	No	33.9	8.1	42.0
Statistics	Chi^2^ = 0.244; df = 1; *p* = 0.62092			
family history of heart disease	Yes	28.5	7.0	35.5
	No	50.5	14.0	64.5
Statistics	Chi^2^ = 0.099; df = 1; *p* = 0.75220			

**Table 7:** Characteristics of participants divided into POL SCORE risk classes and the occurrence of risk factors in the work environment.

Risk Factor in the Work Environment		POL SCORE Risk Class				Total [%]
		Low < 1% [%]	Moderate ≥ 1% and <5% [%]	High ≥ 5% and <10% [%]	Very High ≥ 10% [%]	
stress	Yes	7.8	65.7	22.3	4.2	88.2
	No	14.3	61.9	23.8	-	11.2
Statistics		H = 0.530; *p* = 0.4663				
low physical activity	Yes	10.0	68.0	22.0	-	26.7
	No	8.0	64.2	22.7	5.1	73.3
Statistics		H = 0.942; *p* = 0.3317				
poor diet	Yes	3.7	64.8	27.8	3.7	28.9
	No	10.5	65.4	20.3	3.8	71.1
Statistics		H = 2.144; *p* = 0.1431				

**Table 8:** Characteristics of participants divided into POL SCORE risk classes and the degree of stress occurring in the work environment (*p* = 0.1005).

POL SCORE Risk Class	Assessment of Health Status by Participants			
	None [%]	Low [%]	Medium [%]	Significant [%]
low (<1%)	12.5	12.5	31.2	43.8
moderate (≥1% and <5%)	3.5	23.3	35.3	37.9
high (≥5% and <10%)	7.5	7.5	52.5	32.5
very high (≥10%)	-	14.3	57.1	28.6

## Data Availability

Data are contained within this article.
